# Two-Step Acoustophoresis Separation of Live Tumor Cells from Whole Blood

**DOI:** 10.1021/acs.analchem.1c04050

**Published:** 2021-12-16

**Authors:** Eva Undvall Anand, Cecilia Magnusson, Andreas Lenshof, Yvonne Ceder, Hans Lilja, Thomas Laurell

**Affiliations:** † Department of Biomedical Engineering, Lund University, 221 00 Lund, Sweden; ‡ Department of Translational Medicine, Lund University, 205 02 Malmö, Sweden; § Department of Laboratory Medicine, Lund University, 221 00 Lund, Sweden; ∥ Department of Laboratory Medicine, Surgery (Urology), and Medicine (GU Oncology), Memorial Sloan-Kettering Cancer Center, New York, New York 10065, United States

## Abstract

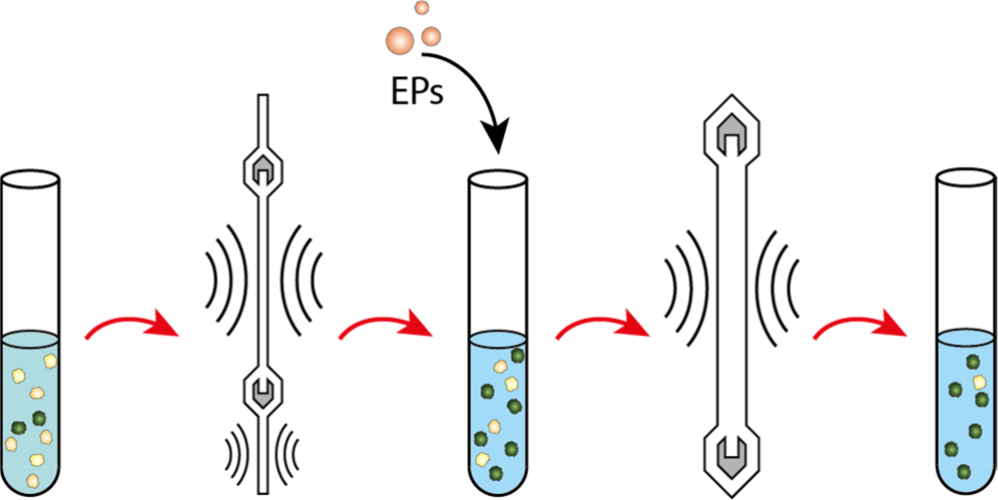

There is an unmet clinical need to extract living circulating tumor cells (CTCs) for functional studies and *in vitro* expansion to enable drug testing and predict responses to therapy in metastatic cancer. Here, we present a novel two-step acoustophoresis (A^2^) method for isolation of unfixed, viable cancer cells from red blood cell (RBC) lysed whole blood. The A^2^ method uses an initial acoustofluidic preseparation step to separate cells based on their acoustic mobility. This acoustofluidic step enriches viable cancer cells in a central outlet, but a significant number of white blood cells (WBCs) remain in the central outlet fraction due to overlapping acoustophysical properties of these viable cells. A subsequent purging step was employed to remove contaminating WBCs through negative selection acoustophoresis with anti-CD45-functionalized negative acoustic contrast particles. We processed 1 mL samples of 1:1 diluted RBC lysed whole blood mixed with 10 000 DU145 cells through the A^2^ method. Additional experiments were performed using 1000 DU145 cells spiked into 1.5 × 10^6^ WBCs in 1 mL of buffer to further elucidate the dynamic range of the method. Using samples with 10 000 DU145 cells, we obtained 459 ± 188-fold depletion of WBC and 42% recovery of viable cancer cells. Based on spiked samples with 1000 DU145 cells, our cancer cell recovery was 28% with 247 ± 156-fold WBC depletion corresponding to a depletion efficacy of ≥99.5%. The novel A^2^ method provides extensive elimination of WBCs combined with the gentle recovery of viable cancer cells suitable for downstream functional analyses and *in vitro* culture.

## Introduction

Although circulating tumor cells (CTCs) in metastatic cancer patients may be exceedingly rare, a noninvasive liquid biopsy can be informative of tumor profile, and CTC enumeration has been clinically validated as a prognostic biomarker predictive of overall survival in advanced cancer stages.^1−4^ Hence, detectable or higher counts of CTC in blood is significantly associated with poor outcomes.^1−3,5^ Molecular characterization of enriched CTC populations can provide information on therapeutic targets and drug resistance mechanisms.^5,6^ Recently, androgen receptor splice variant 7 (AR-V7) CTC-testing was clinically validated to facilitate decision-making of androgen receptor (AR) signal inhibitor therapy for men with metastatic castration-resistant prostate cancer (mCRPC), which has poor prognosis.^7^ Only a minute subset of cells shed from a primary tumor into the bloodstream survive the shear stress and lack of cell-to-cell adhesion within blood vessels to manifest tumor-initiating capacities at a distant location after extravasation.^8^ The details of the metastatic process are to a large extent unknown; therefore, the recovery of viable CTCs for downstream analysis is of major interest.

The scarcity of CTCs entails the necessity of a purification procedure to discriminate the malignant cells from other nucleated cells in blood. Therefore, it is critical to explore novel means to isolate CTCs, such as label-free acoustophoresis, as commercially available CTC isolation assays, e.g., CELLSEARCH and Adnagen, depend on capture using antibodies against the epithelial cell adhesion molecule (EpCAM). This limits CTC detection to EpCAM positive cells while excluding approximately 60% of the entire CTC population that are either EpCAM negative or have low EpCAM expression.^9^ The precise control of cells using microfluidics have emerged in several CTC technologies with diverse approaches for cell enrichment, including passive methods like deterministic lateral displacement (DLD),^10^ inertial separation,^11,12^ and mechanical antibody-coated microstructures,^13,14^ as well as active force methods like dielectrophoresis (DEP),^15−20^ or antibody-dependent magnetophoresis.^21,22^ However, CTC assays frequently use a combination of selection approaches, often including positive selection with monoclonal antibodies.^10,13,14,23^

Moreover, most of the highly specialized technologies aimed at CTC purification are designed for fixed cells and not for viable cells with intact tumor-initiating potential. Enrichment of fixed cells has some technical advantages, as the majority of the cellular proteins and peptides become crossed-linked and thereby resistant to degradation or deformation. This enables a longer sample processing window and transportation of samples between hospitals and clinical labs. It also facilitates intracellular staining of markers of interest, such as hormonal receptors, specific enzymes, and cytokeratins. The enrichment of viable cells may be less permissive to preanalytical challenges, such as transport conditions but be compatible with the abovementioned downstream staining processes as well as genomic analyses through, e.g., fluorescence *in situ* hybridization (FISH) or whole genome sequencing (WGS). However, a critically important advantage of live cell enrichment is the feasibility to pursue functional studies and *in vitro* culturing of patient-derived malignant cells. Therefore, recent advances in cell culture technologies and the major achievement of establishing new cell lines using CTCs have opened a new path for CTC research.^24,25^ An *in vitro* expansion of captured tumor cells could importantly facilitate personalized medical advances and enable drug screening, predict therapeutic responses in individual patients, and help to identify cellular characteristics key to initiating metastatic lesions.^24^

A key prerequisite to establishing a patient-derived CTC cell line is to isolate intact viable cells. Acoustophoresis holds promise as an efficient enrichment method for viable CTCs due to its inherently gentle, noncontact, label-free, and high throughput microfluidic approach.^26,27^ The method uses acoustic forces to manipulate and sort cells and particles within a resonant microchannel. Positional displacements depend on the acoustic mobility of cells and smaller particles, where size is a major factor, as the acoustic radiation force scales with particle volume. Previous studies with acoustic cell separation highlighted the problem of overlapping acoustic properties of small epithelial cancer cells and certain subpopulations of white blood cells (WBCs), such as densely granular granulocytes, in particular, eosinophilic cells.^27^ For cells not subjected to fixation treatments, the overlap is even more substantial, with additional contamination of granulocytes in the diverted cancer cell fraction.^27^ Therefore, a second purification step may be necessary to remove contaminating cells to obtain a sufficiently high fraction of cancer cell purity.

There are several approaches for live cell WBC depletion, such as negative acoustic contrast particles,^28^ magnetic beads,^23^ density medium iso-acoustic focusing,^29^^29^ or density gradient, e.g., RosetteSep.^24^ A density gradient centrifugation process might result in lower recovery of cancer cells as larger CTCs and CTC clusters tend to sediment along with the red and white blood cells or migrate into the plasma fraction.^30,31^ Loss of CTC viability has also been suggested to be the result of cytotoxicity of density mediums.^32^ The requirement for the magnetic bead separation Dynabeads is a depletion efficacy of >85% (Invitrogen) of target cells. This is significantly lower than the depletion efficacy of >98% accomplished by anti-CD45-functionalized negative acoustic contrast elastomeric particles (EPs)^28^ or by acoustophoresis alone, which deplete 95% of the viable WBCs or 99.5% of paraformaldehyde (PFA) fixed WBCs.^27^ Furthermore, the previously reported proof-of-concept study of negative acoustic contrast particle immuno-acoustophoresis displayed tumor cell recoveries between 85 and 90%.^28^

Biofunctionalized negative acoustic contrast particles were first used to transport polystyrene particles to pressure antinodes in a microfluidic channel in the absence of flow.^33^ A later study captured and immobilized cells at the antinodes at the walls of the microchannel.^34^ By employing continuous flow, separation and sorting of immuno-functionalized negative contrast particles and positive contrast cells were demonstrated.^35^

In this paper, we have developed a two-step sequential acoustophoresis (A^2^) method to isolate viable cancer cells from red blood cell (RBC) lysed whole blood. The two steps are based on the acoustic translocation of cells and particles, first through a primary separation step, followed by a secondary purging step to remove contaminating WBCs by negative selection acoustophoresis. In the second step, a 3λ/2 acoustic standing wave configuration is employed, which also locates pressure antinodes at a distance of λ/2 from the sidewalls to reduce the risk of EPs being trapped at the pressure antinodes along the sidewalls.^36^ The benefits of performing preseparation acoustophoresis prior to WBC depletion by EPs are multiple, including cost-efficacy, as fewer EPs and antibodies are needed, and performance-based process as the starting cell density will decrease considerably, reducing hydrodynamically linked carryover. Further, we demonstrate the biocompatibility of the A^2^ method through viability and proliferation studies of the recovered cancer cells.

## Experimental Section

### Cell Culture and Healthy Blood Donors

Human prostate cancer cell line DU145 and breast cancer cell line MCF7 were obtained from American Type Culture Collection (ATCC). Cells were cultured in Roswell Park Memorial Institute (RPMI) 1640 medium (Sigma-Aldrich, Schnelldorf, Germany), and Dulbecco’s modified Eagle’s medium (DMEM) (Sigma-Aldrich) supplemented with 10% fetal bovine serum (FBS; Sigma-Aldrich), 55 units mL^–1^ penicillin and 55 μg mL^–1^ streptomycin (Sigma-Aldrich). The cells were cultured at 37 °C under a 5% CO_2_ atmosphere and harvested at 80% confluency. Blood was collected from healthy volunteers at the Hematology Department at Skåne University Hospital (Lund, Sweden) after written informed consent, in accordance with the Helsinki Declaration and after being approved by the local ethics committee. Blood was collected in Vacutainer tubes (BD Bioscience, Temse, Belgium), containing ethylenediaminetetraacetic acid (EDTA) and subjected to isotonic lysis (according to manufacture’s instructions) to remove red blood cells by BD Pharm Lyse (BD Biosciences) or BD FACS lysing solution for PFA fixed cells. Blood was processed and used for experiments on the day of collection.

### Synthesis and Activation of Biotinylated EPs

Polydisperse biotinylated EPs were synthesized as previously described,^28,37^ and detailed information can be found in the Supporting Information. To bind nonfixed WBC, the size fractionated EPs were functionalized with streptavidin conjugated mouse anti-human CD45 monoclonal antibody clone MEM-28 (Sigma-Aldrich).^28^ Following functionalization, the particles were washed twice and finally resuspended in FACS buffer (1× Dulbecco’s Phosphate-Buffered Saline [DPBS], 1% FBS, 2 mM EDTA), or FACS buffer containing 0.1% Pluronic F-108 surfactant (FACS buffer P).

### Immunostaining, Flow Cytometry Enumeration, and ImageStream Analysis

The concentration of synthesized antibody-activated EPs was determined by flow cytometry FACS Canto II (BD Biosciences) FSC vs SSC scatter plots, prior addition to the primary separation sample output. The concentration of DU145 and MCF7 cancer cells for spiking was evaluated in a similar way, with the addition of a fluorophore. Detailed labeling protocol can be found in the Supporting Information. Flow cytometry was also used to compare the cell separation outputs by analyzing central and side outlet fractions and to enumerate recovered cancer cells and any contaminating WBC in the final central output fraction. Examples of the gating strategy can be found in Figure S1.

Samples of 0.5–1.0 mL RBC lysed blood spiked with 1000–10 000 DU145 cells were stained with anti-EpCAM clone EBA-1 (BD Biosciences), and WBCs were labeled with anti-CD45 clone HI30 (BD Biosciences). Amnis ImageStream Mk II (Millipore, Burlington, MA) was used to obtain images of EP-complexes with captured WBCs stained with DAPI (Sigma-Aldrich) and of isolated DU145 cells stained with EpCAM. DAPI was used to stain WBCs when EPs were present, as the anti-CD45 surface marker normally used for WBC staining was also used by the EPs.

### Setup of Primary Separation Step—Separation of Fixed vs Viable Cells

**Figure 1:**
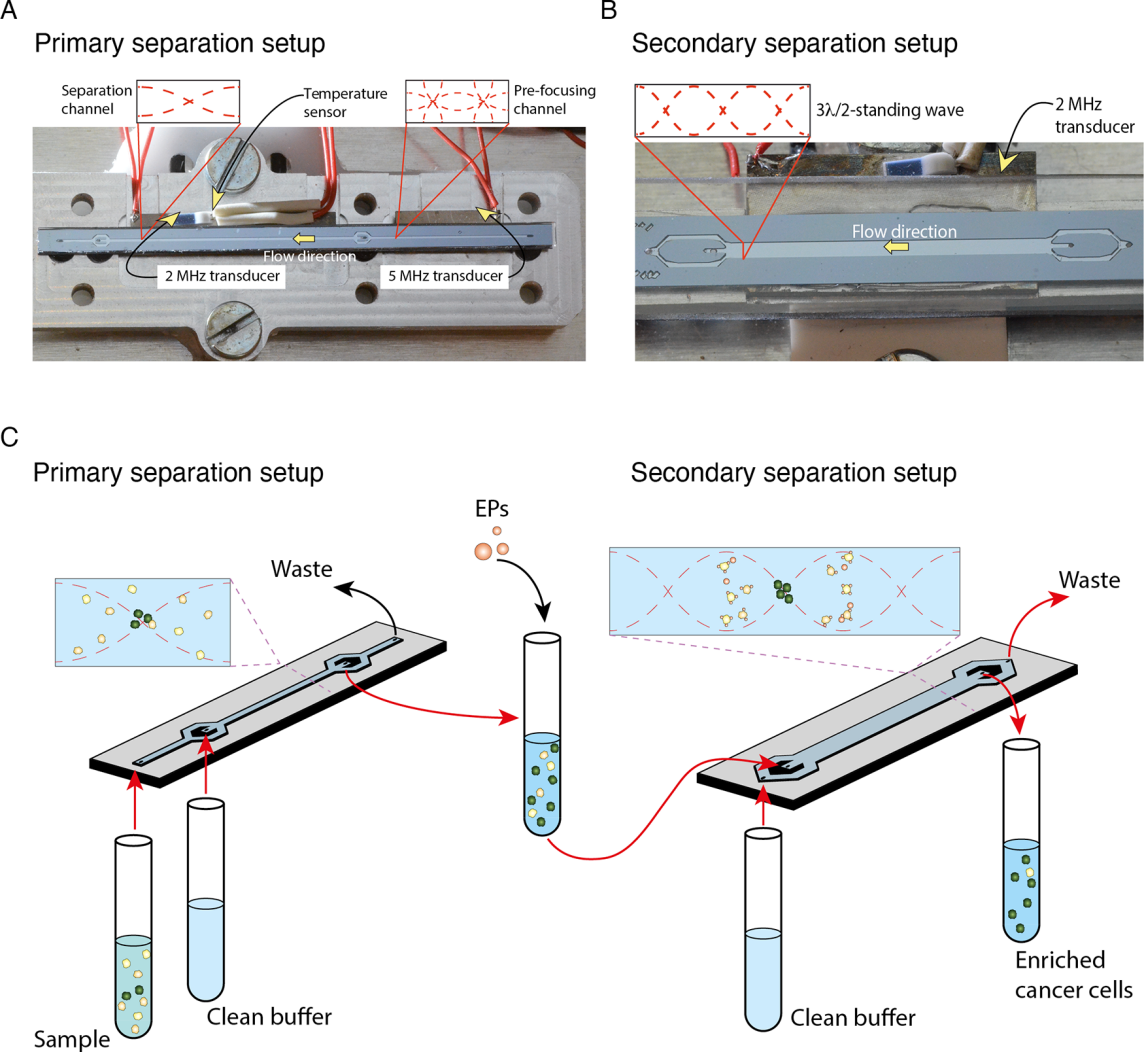
Overview of the of two-step acoustophoresis (A^2^). (A) Photograph of the primary separation chip and aluminum chip holder prior to assembly. Showing the prefocusing channel followed by the separation channel, the two piezoelectric transducers for sound generation, as well as a temperature sensor. (B) Photograph of the multinode (3λ/2) purging chip with one piezoelectric transducer and separation channel. (C) Schematic of the workflow and separation principle in A^2^. In the primary separation setup, a cell sample input represented by white (WBCs) and green (cancer cells) circles enters the chip through the prefocusing channel. After passing through the separation channel, the cells are collected at the central outlet. The cells are incubated with elastomeric particles (EP) and subsequently processed through the secondary multinode separation chip. The purified cancer cell fraction is collected at the central outlet.

The mechanism of separating cultured cancer cells from WBCs by acoustophoresis has previously been described in Augustsson et al.,^38^ and the primary cell separation step has previously been described in Magnusson et al.^27^ Briefly, the chip was manufactured in silicon and glass using previously reported microfabrication processing^39^ with an initial prefocusing channel (length × width × depth; 20 mm × 300 μm × 150 μm), where cells/particles are exposed to acoustic radiation forces at 5 MHz (4.890 MHz), acting transversely to the flow in two dimensions (Figure 1A). As cells enter the subsequent separation channel (30 mm × 380 μm × 150 μm), the cells are laminated in the proximity of the channel sidewalls by the introduction of cell-free medium (FACS buffer), through the central branch of a trifurcation inlet at the beginning of the separation channel. In the separation channel, cells are exposed to a 2 MHz (1.980 MHz), half-wavelength acoustic standing wave field that forces them toward the center of the separation channel. The force is dependent on cell size, shape, density, and compressibility, causing larger cells to migrate faster than smaller cells toward the channel center. Consequently, the majority of the cancer cells can be collected through the central branch of a trifurcation outlet while the smaller WBCs are discarded as they exit through the side branches at the end of the separation channel. Samples of 200 μL containing approximately 2000 DU145 cells and 300 000 WBCs (fixed or nonfixed cells) that were fluorescently labeled were processed at a flow rate of 75 μL min^–1^, together with a central sheath flow of 300 μL min^–1^. For PFA fixed cells, 4% PFA was used and incubated with cells for 25 min at room temperature. Cells were washed with DPBS after fixation.

### Setup of Secondary Separation Step

The second purging step reused an acoustophoresis chip design from an earlier study.^36^ In short, the microchannel (23 mm × 1125 μm × 150 μm) has a trifurcation inlet design with central sample and side buffer inlets (Figure 1B). The outlet design is analogous, with a central sample outlet. The 1125 μm wide channel combined with an actuation frequency of 2 MHz (1.878 MHz ± 50 kHz), matching three half-wavelengths (3λ/2), translates into a centered positioned pressure node and two bordering pressure antinodes λ/2 from the sidewalls, followed by two additional pressure nodes and adjacent antinodes at the sidewalls of the channel. The 3λ/2 configuration confines the EPs to the two central antinodes, located 375 μm from the sidewalls, and thus reduces the risk of EP trapping at the pressure antinodes along the sidewalls.^36^ Frequency modulation of ±50 kHz with a scan rate of 200 kHz ms^–1^ was employed to avoid EP’s aggregation at a Gor’kov potential minima.^40^ An approximately 40-fold excess of antibody-activated EPs to the remaining contaminating WBC was added to the central output sample from the primary step. The sample was incubated with continuous rotation in the dark for 1 h at room temperature. After incubation, the sample was processed through the central inlet of the 3λ/2 acoustophoresis chip at 100 μL min^–1^, together with a side sheath flow of 400 μL min^–1^, ensuring that all input samples were confined within the two central antinodes. A schematic image of the workflow and separation can be found in Figure 1C.

### Cell Size Assessment

Cell counts and cell size analysis were performed by Coulter counter using RBC lysed blood and DU145 cancer cells for both live and fixed cells. Whole blood was lysed and washed as described earlier and resuspended in FACS buffer. DU145 cells were harvested and resuspended in FACS buffer as reported. For fixing cells, 4% fresh PFA was used. All cells were kept on ice prior to the Coulter counter analysis. FACS buffer was used for background measurements. The Coulter counter measures the change in electrical impedance when a cell passes through an orifice, which is recorded as a voltage pulse that is proportional to the volume displaced by the cell.

### Optimal WBC Cell Concentration

A series of 0.5 mL samples with increasing concentrations of WBCs (1.0 × 10^5^ to 6.0 × 10^6^ cells mL^–1^) were run through the primary acoustic separation chip using a constant amount of spiked DU145 cells (1.0 × 10^4^ cells). Cells were immunofluorescently labeled with anti-CD45-APC and anti-EpCAM-PE to enable flow cytometry enumeration. Central and side outlet cell fractions were analyzed by flow cytometry, as previously described.

### Analytical Validation of the A^2^ Method for Live Cell Separations

During acoustic cell separations, we had previously used FACS buffer,^38^ whereas negative acoustic selection was previously performed using a buffer containing 0.1% Pluronic surfactant.^28^ To streamline the A^2^ method, we evaluated the performance characteristics of the cell separation in the primary step using FACS buffer compared with that of Pluronic surfactant added to the FACS buffer. Next, we compared the use of FACS buffer only throughout the complete A^2^ method experiment vs using FACS buffer in the primary separation step with the subsequent addition of 10 μL 10% Pluronic F-108 into the 1 mL sample output prior to the addition of EPs. As previously reported, the final concentration of Pluronic F-108 in the secondary step was 0.1% (FACS buffer P).

The analytical validation of the A^2^ method was performed using 1 mL samples containing 1:1 mixed RBC lysed whole blood and buffers, as described above, spiked with 10 000 fluorescently labeled DU145 or MCF7 cells. The samples were run in triplicate for each buffer, and the experiment was repeated in triplicate. The final output samples were analyzed and enumerated with FACS Canto II (BD Biosciences).

### Spiking of 1000 DU145 Prostate Cancer Cells

One thousand DU145 cells (estimated by flow cytometry concentration measurement) were labeled as previously described and spiked into 1 mL FACS buffer containing 1.5 × 10^6^ WBCs from RBC lysed whole blood before being processed with the A^2^ method. The final central output sample was enumerated for cancer cell number and the remaining WBCs, by flow cytometry analysis. The experiment was repeated in triplicate.

### Cell Proliferation and Viability

To investigate whether the viability of the cancer cells was negatively affected by the acoustic separation process, we subjected 1 mL cell samples (3.0 × 10^6^ DU145 cells) to the primary cell separation step, followed by 1 h rotating incubation at room temperature with or without EPs present, before processing the secondary separation step. The cell concentration of the processed sample was estimated by flow cytometry, and 300 000 cells per well were seeded in a 6-well plate for subsequent culture. Control cells (cells incubated on ice during the length of the experiment and not subjected to acoustophoresis or EPs incubation) were seeded simultaneously. After 3–5 days, the cells were harvested and either passaged for further culture (until passage 3) or stained with 7AAD (Sigma-Aldrich). Flow cytometry was used to analyze 100 000 cells to estimate the percentage of dead cells (7AAD^+^). The experiment was run in triplicates and repeated three times.

## Results and Discussion

### Primary Acoustophoretic Separation of Fixed vs Live Cancer Cells from WBC

**Figure 2:**
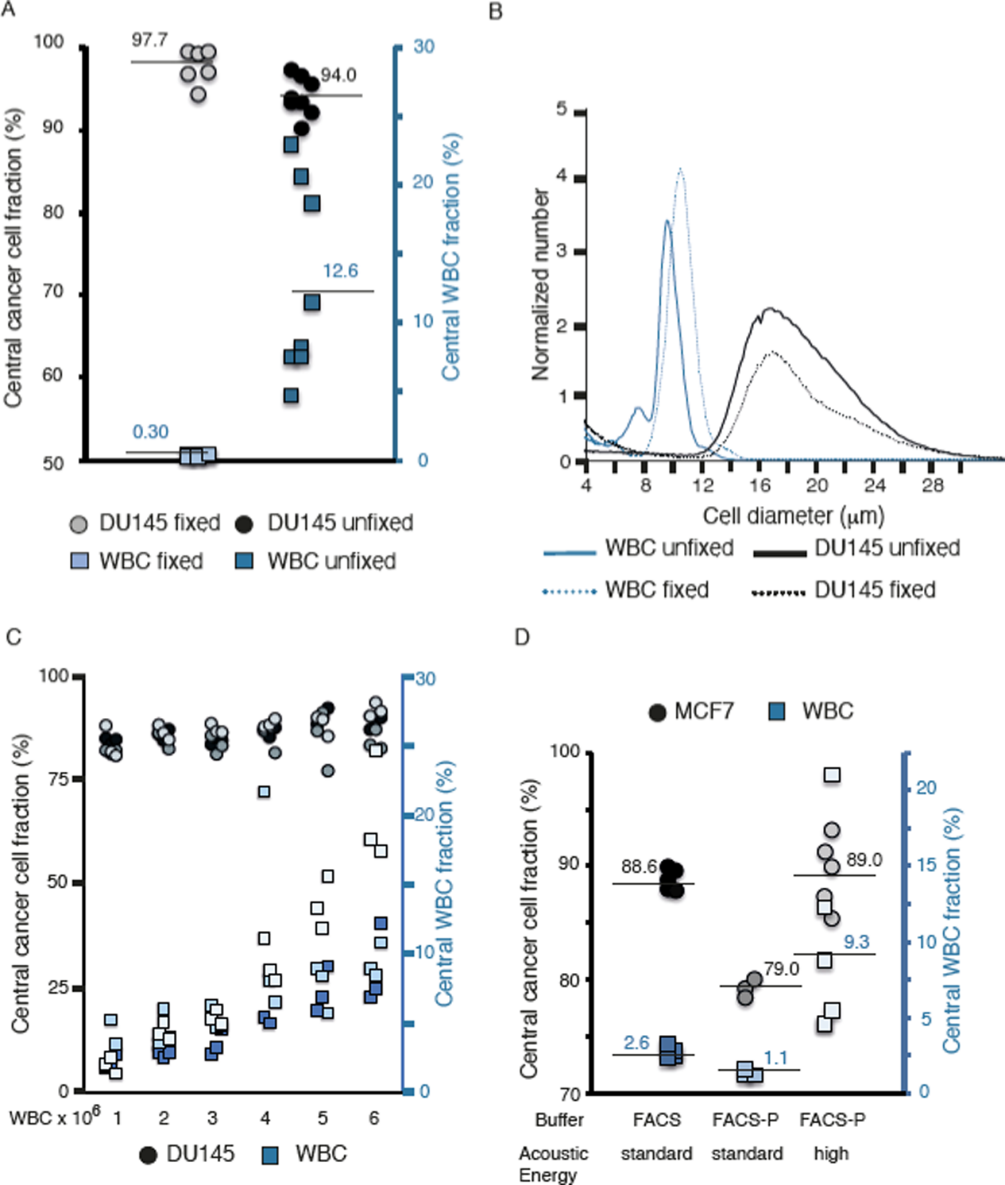
Characterization for optimal cell separation by acoustophoresis. (A) Comparison of cell separation by acoustophoresis of PFA fixed vs unfixed DU145 cells and WBCs. (B) Cell size measurements by Coulter counter of PFA fixed and unfixed WBC and DU145 cells. The graph shows the result from a representative experiment out of the three performed experiments. (C) Central outlet cancer cell and WBC fractions vs total cell concentration. A series of samples with increasing concentrations of unfixed WBCs (1.0 × 10^6^ to 6.0 × 10^6^ mL^–1^) was run through the primary separation chip using a constant concentration of spiked unfixed DU145 cells (1.0 × 10^4^ mL^–1^). Three different experiments with *n* = 3 replicates were performed. Each experiment is color-coded in different shades of gray (DU145 cells) and blue (WBCs). (D) Comparison of unfixed cell separation: MCF7 cells (circles) and WBC (squares) in different buffers, FACS buffer vs FACS buffer containing Pluronic (FACS-P).

In the primary acoustic separation step, we found that the separation of unfixed, viable WBCs from DU145 cancer cells was less efficient compared with that of PFA fixed cells (Figure 2A). Approximately 2000 fixed DU145 cells were spiked into fixed RBC lysed blood, of which 97.7% of the cancer cells could be focused to the central outlet, with 0.3% of the WBCs contaminating the cancer cell fraction. Analogously, 2000 unfixed DU145 cells were spiked into unfixed RBC lysed blood, in which 94.0% of the DU145 cells focused to the central outlet, with an average WBC contamination of 12.6%. One explanation why there were lower recoveries of unfixed cancer cells compared with fixed cancer cells could be due to the loss of a larger dead cell population in the unfixed cell separation. Dead cells have a different acousto-mechanical phenotype as to that of live cells,^41^ which can explain the less efficient focusing of dead cells (Figure S2) in the acoustic field.^42,43^ This emphasizes the need for a secondary purging step when aiming for minimal WBC contamination when acoustophoresis is used to isolate high recovery live cancer cells.

### Cell Size Assessment

We hypothesized that smaller differences in cell size between unfixed cells (WBCs vs DU145) as compared with PFA fixed cells (WBC vs DU145) contributed to the challenges of separating unfixed cells. The primary acoustic radiation force (*F*_R_, eq 1) is the main force acting on the cells in free flow acoustophoresis, where the radius scales to the power of three (*r*^3^), and is therefore a major contributor to acoustophoretic velocity differences between cells.^40^
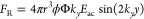
1where *r* is the particle radius, Φ is the acoustic contrast factor, *k_y_* = 2π/λ is the wavenumber, *E*_ac_ is the acoustic energy density, and *y* is the distance from the wall.

However, the Coulter counter measurements demonstrated larger differences in cell size between unfixed cells (WBCs vs DU145) as compared with fixed cells (Figure 2B). Additionally, the overlap in size distribution for fixed cells (WBCs vs DU145) is more extensive as compared with unfixed cells. As this would further impair the separation of fixed cells, we concluded that the difference in size between WBCs and DU145 cells does not explain the observed superior separation of fixed cells. Therefore, differences in density and compressibility of unfixed cells may provide a stronger impact on the separation performance than size distributions alone.

We also found that Coulter counter data showed wider size distribution of unfixed WBCs (6–11 μm) compared with fixed WBCs (8–12 μm). Thus, fixation generates a more homogeneous size for the different blood cells.

### Optimal WBC Cell Concentration

Due to hydrodynamic interactions between closely positioned cells in a suspension,^44^ separation techniques that rely on force fields (e.g., magnetic, acoustic, electric) acting on cells in suspension display a dependency on sample cell concentration in relation to separation performance. The normal concentration of WBCs in blood exceeds that where hydrodynamic interaction plays a role, and it is therefore necessary to dilute clinical samples. We found that the maximum cell concentration for nonfixed cells was approximately 3.0 × 10^6^ cells mL^–1^ (Figure 2C). This is similar to the cell concentration (∼3.5 × 10^6^ cells mL^–1^) previously reported for PFA fixed cells that can be processed without compromising acoustophoretic cell separation performance, as higher cell concentrations lead to increased WBC contamination in the central outlet fraction.^27^

### Optimizing Buffer Conditions

**Figure 3:**
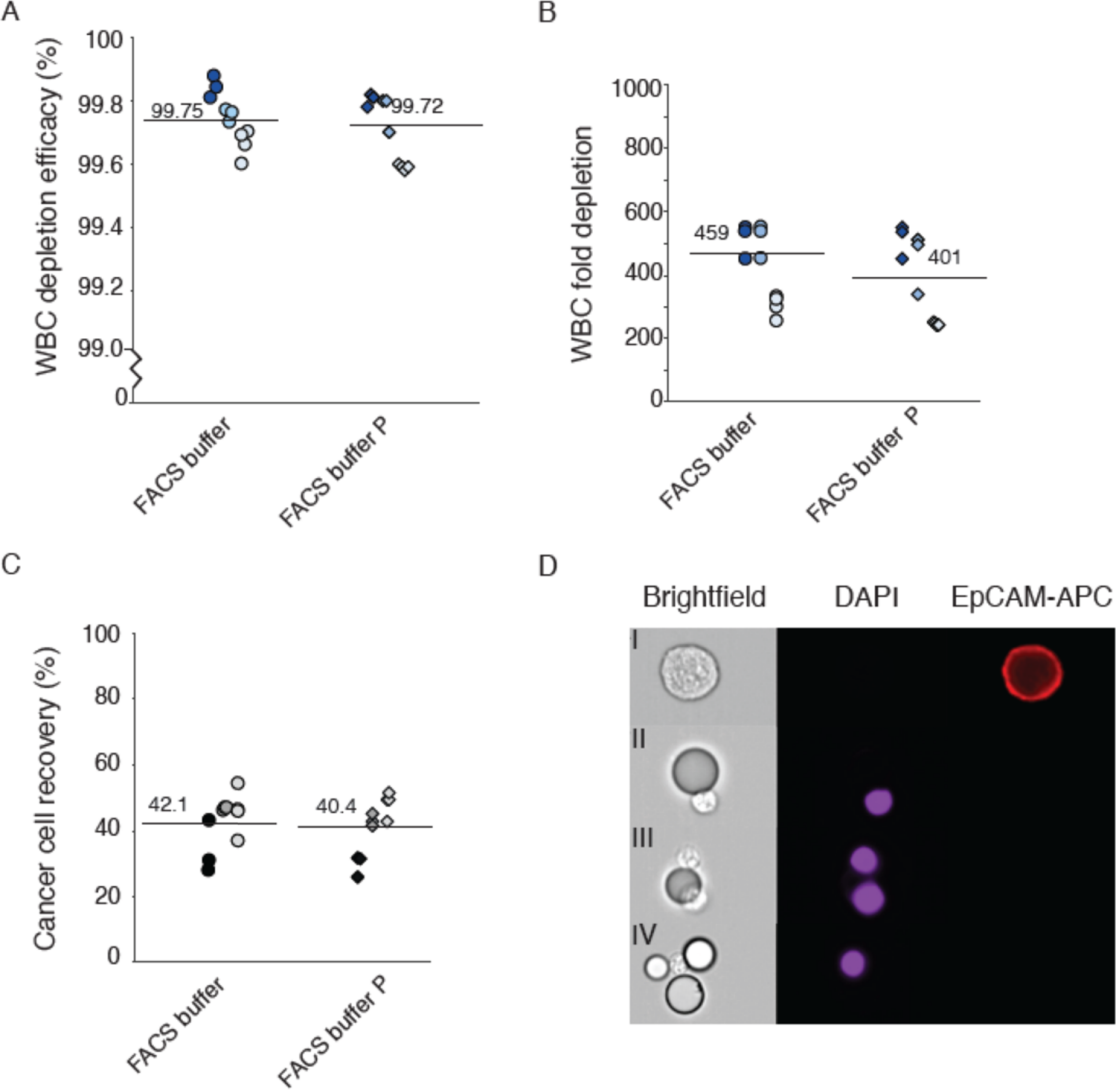
Negative depletion of WBCs with elastomeric particles. (A) WBC depletion efficiency and (B) WBC fold depletion after the two-step acoustophoresis (A^2^) of cancer cells with or without Pluronic in the buffer. (C) Cancer cell (DU145) recovery after A^2^ with or without Pluronic in the buffer. The graphs show three experiments (*n* = 3) with blood from three different healthy donors. (D) ImageStream DU145 cell stained with anti-EpCAM-APC (I), one WBC stained with DAPI bound to an elastomeric particle (II). Two DAPI stained WBCs bound to the same elastomeric particle (III). One DAPI stained WBC bound to three elastomeric particles (IV).

Previously, the acoustophoretic cell separation has been performed in FACS buffer,^27^ whereas a Pluronic F-108-containing buffer (FACS buffer P) was used during the proof-of-principle work of negative acoustic contrast particle acoustophoresis.^28^ As it is desirable to use one buffer throughout the entire A^2^ separation process, we evaluated the optimal A^2^ buffer composition. First, we evaluated the separation performance of unfixed WBC and MCF7 cells in FACS buffer (*n* = 5) vs FACS buffer P (*n* = 3 + 5) in the primary separation step. Using the FACS buffer P, we found that fewer MCF7 cells were focused in the microchannel and collected at the central outlet at the same piezo actuation voltage as compared with FACS buffer (Figure 2D, standard acoustic energy). To obtain similar cancer cell recovery (88.6%) with FACS buffer P as that obtained using FACS buffer, there was a close to 4-fold increase in WBC contamination (from 2.6 to 9.3%), indicating that FACS buffer P was suboptimal for the initial separation step (Figure 2D, high acoustic energy). Subsequently, we compared the use of FACS buffer throughout the A^2^ experiment (with unfixed WBCs and DU145 cells) with FACS buffer used during step 1, followed by 0.1% Pluronic F-108 buffer during the intermediate EP incubation phase and the final secondary purging step. We found no difference in cancer cell recovery or WBC fold depletion by the addition of a buffer surfactant and therefore concluded that FACS buffer can be used throughout the A^2^ procedure (Figure 3A,B). As our previous report^27^ showed, acoustic focusing of various epithelial cancer cell lines are comparable and interchangeable in analytical validation and holds promise for successful enrichment of CTCs from different epithelial carcinomas.

### Analytical Validation of the A^2^ Method for Live Cell Separation

Analytical validation of live cell separations with A^2^ acoustophoresis, with a primary acoustophoresis step combined with a secondary WBC purging step by immuno-activated negative acoustic contrast particles, showed a depletion efficacy of over 99.7 ± 0.1% (Figure 3A), which corresponds to a WBC fold depletion between 401 ± 140-fold and 459 ± 188-fold (FACS buffer 0.1% P and FACS buffer, respectively). The recovery of the 10 000 spiked DU145 cells was between 40.4 ± 9.6 and 42.1 ± 7.0% (Figure 3B,C). Thus, 1 mL samples with whole blood and FACS buffer (1:1) (with a maximum concentration of 3.0 × 10^6^ cells mL^–1^) had after the A^2^ process a final contamination of approximately 5000 WBCs.

A major contributing factor to the inter-variability between the experiments is due to the use of blood from different donors. Unfixed blood samples displayed a wider size distribution of the different WBC subpopulations compared with fixed blood samples, as PFA treatment provided more uniform sizes, as discussed above (Figure 2B) and also reported by Urbansky et al.^45^ Any variability in the proportion of lymphocytes, monocytes, and granulocytes will affect the size distribution and acoustic mobility of the WBC population in a blood sample. Additionally, both cultured cancer cells and CTCs in clinical samples vary in cell size.^46−48^ Hence, we would anticipate that experiments using smaller-sized cancer cells spiked into WBCs with a wider size distribution would lead to lower cancer cell recovery with higher WBC contamination. Intra-variability between replicates could likely be caused by varied separation efficiency of the primary separation step at higher WBC concentrations as well as by flow disturbances due to cell aggregates in the secondary purging step.

Figure 3D shows representative images from Amnis ImageStream of elastomeric particle-white blood cell (EP-WBC) complexes, where an anti-CD45 immuno-functionalized EP can bind to one (Figure 3D panel II) or more (Figure 3D panel III) WBCs (DAPI nuclear counterstain, purple). Also, a single WBC can bind to several functionalized EPs (Figure 3D panel IV). DU145 cells showing EpCAM (red) expression do not bind EPs (Figure 3D panel I).

### Spiking of 1000 DU145 Prostate Cancer Cells

**Figure 4:**
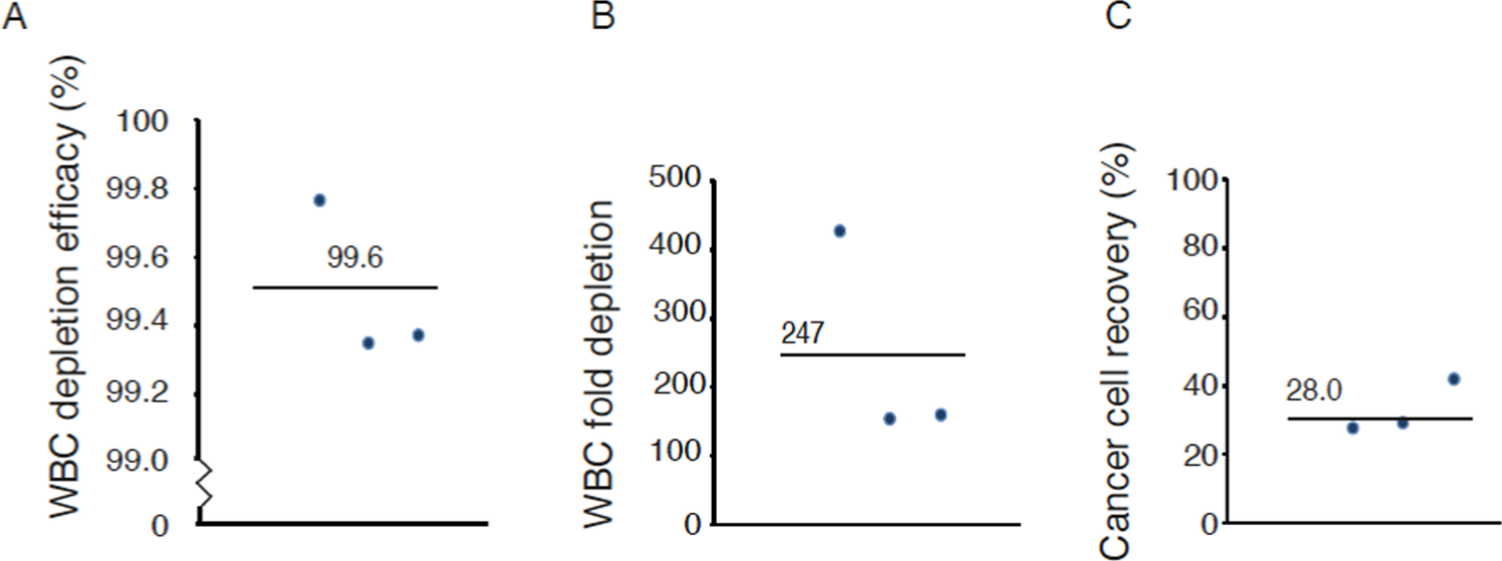
Validation of two-step acoustophoresis (A^2^) with 1000 spiked cancer cells. (A) WBC depletion efficiency, (B) WBC fold depletion, and (C) cancer cell (DU145) recovery after A^2^. The plots show three repetitive experiments with blood from three different healthy donors.

The performance of the A^2^ method was investigated in spiking experiments with a smaller number (1000) of DU145 cells. Here, the A^2^ method displayed a 99.6 ± 0.2% depletion efficacy (Figure 4A), generating a 282 ± 177-fold WBC depletion and a 28.0 ± 0.5% DU145 cell recovery (Figure 4B,C). Thus, 276–285 out of 1000 spiked in DU145 cells were recovered in the three experiments and with a final contamination of approximately 6600 WBCs from the 1 mL sample with ≈1.5 × 10^6^ WBCs.

Similar to what was discussed above in reference to the primary acoustic separation step, differences in the composition of the WBC population can also influence the success of the secondary purging step. Although CD45 is the most common marker in WBCs, its expression level varies widely between different WBCs, which affects the depletion performance of anti-CD45 immuno-functionalized EPs.^10^ Including additional markers targeting granulocytes could be a solution for increased fold depletion of the leukocytes. However, remaining WBCs do not usually limit the downstream *in vitro* and *in vivo* functional studies.^25^

### Cell Proliferation and Viability

We have previously shown that the viability and function of cancer cells are not detectably affected by acoustophoresis.^12,25^ Numerous measures, such as cell survival, proliferation, and prostate-specific antigen (PSA) secretion, have been investigated, and acoustophoresis showed no significant effect on any of the tested factors.^26,38^ However, previous studies were limited to a single acoustic separation step, whereas cancer cells processed with the present A^2^ method were subjected to two consecutively acoustophoretic steps and a rotating incubation step in between runs. Therefore, we assessed the viability and proliferative ability in triplicate to determine whether the cancer cells manifested any harmful effects from being subjected to sequential acoustophoresis.

**Figure 5:**
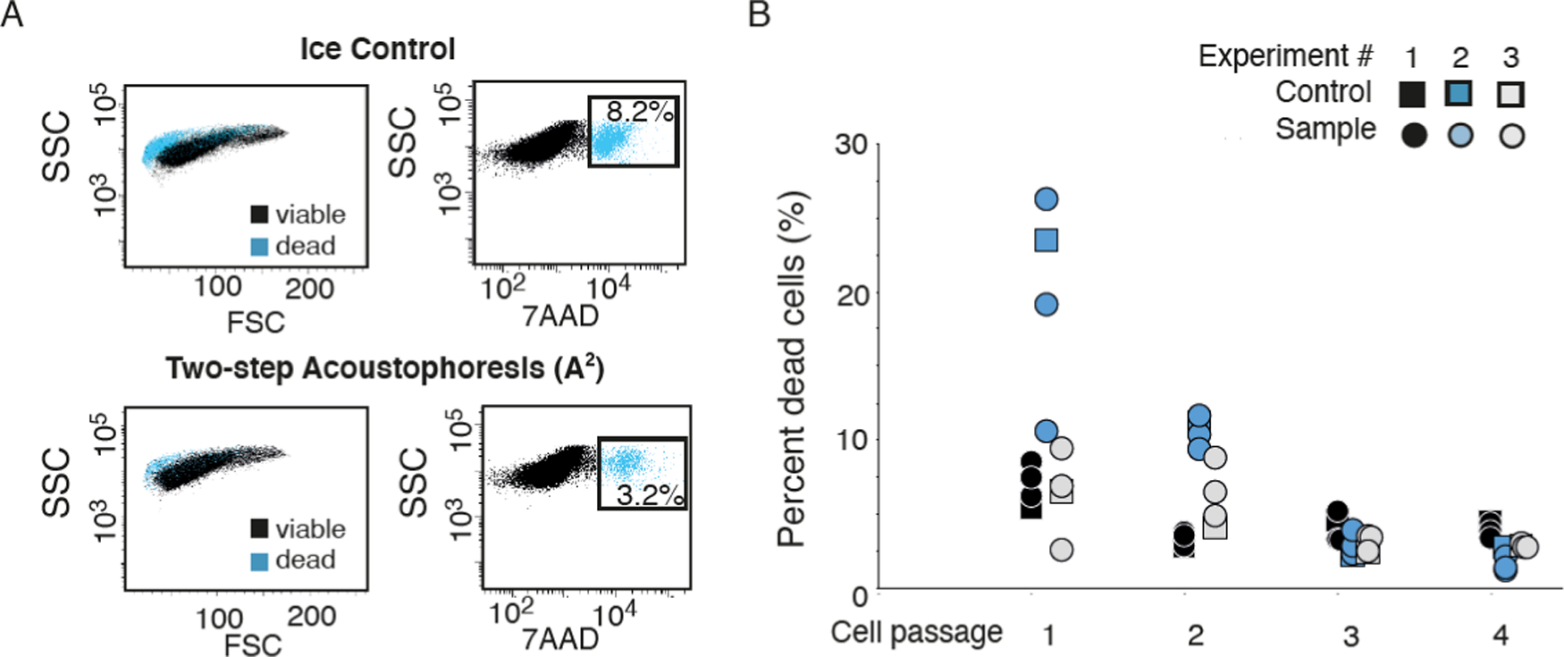
Cell viability and acoustophoresis. (A) Representative flow cytometry plots showing FSC/SSC (left) and 7AAD/SSC (right) for DU145 cells after incubation on ice for 3 h (top panel) and after the two-step acoustophoresis (A^2^) procedure (lower panel), for 50 000 measured cells (*n* = 3). (B) Percentage dead cells (DU145) shown after the A^2^ procedure and culturing in standardized cell incubator and culture medium, after 1–4 cell passages (circles), and after control ice incubation (squares). After each cell passage, 300 000 cells were seeded in each well. The plot shows three different experiments (*n* = 3).

DU145 cells were stained with 7AAD after the second purging step, which showed that 3.2% of the 100 000 enumerated DU145 cells were dead based on flow cytometer analysis. Compared with 8.2% of the control cells (only incubated on ice throughout the experiment) stained positive for 7AAD (Figure 5A). That the tumor cell fraction collected after the A^2^ process contained a smaller number of dead cells compared with control cells incubated on ice can likely be due to the fact that most dead cancer cells present in the original cell suspension did not focus to the central outlet in the acoustic field (Figure S2) and therefore were removed in the primary acoustic separation step. This indicates that the most critical aspect of cell survival is the processing time rather than A^2^ separation.

Cancer cells seeded for culture after the A^2^ separation procedure as well as the control sample incubated on ice showed no difference in percentage dead cells after passage 1–4 (Figure 5B). The re-cultured cells attached to the well bottom and proliferated equivalently. The initial percentage of dead cells was higher in experiment 2 (blue circles) compared with experiment 1 (black circles) and 3 (gray circles), which might be explained by that cancer cells used in experiment 2 were left out of culture for a longer time period. Again, this indicates that the time factor is much more important for cell viability compared with subjecting the cells to ultrasound and shear forces in the microchannels. The presence of EPs had no effect on the viability of re-cultured cancer cells as they were washed away through the passages.

## Conclusions

In this study, we report a novel two-step acoustophoresis method (A^2^) for the isolation of viable cancer cells from RBC lysed whole blood. The two steps are based on acoustic translocation of cells and particles, first through a primary acoustophoresis separation step based on the intrinsic acoustophysical properties of the cells, followed by a secondary purging step to deplete the contaminating WBCs by negative selection using anti-CD45 immuno-functionalized elastomeric particles. This method delivers viable cancer cells for further downstream analysis and growth *in vitro*. We believe that this label-free, noncontact, and gentle approach holds promise to obtain live CTCs from clinical samples for subsequent culturing and functional assays, enabling personalized medical treatment strategies.

## Supporting Information Available

Flow cytometry gating strategy for concentration measurement of samples; separation data of viable and dead cells by acoustophoresis at two different acoustic energy levels (PDF)

## Abbreviations

A^2^: two-step acoustophoresis
AR: androgen receptor
AR-V7: androgen receptor splice variant 7
ATCC: American type culture collection
DMAP: 4-(dimethylamino)pyridine
DMEM: Dulbecco’s modified Eagle’s medium
DPBS: Dulbecco’s phosphate-buffered saline
DSC: *N*,*N*′-disuccinimidyl carbonate
EDTA: ethylenediaminetetraacetic acid
EpCAM: epithelial cell adhesion molecule
EPs: elastomeric particles
FBS: fetal bovine serum
FISH: fluorescence *in situ* hybridization
mCRPC: metastatic castration-resistant prostate cancer
PFA: paraformaldehyde
PSA: prostate-specific antigen
RBCs: red blood cells
RPMI: Roswell Park Memorial Institute Medium
WBCs: white blood cells
WGS: whole genome sequencing

## References

[ref1] Cristofanilli M.; Budd G. T.; Ellis M. J.; Stopeck A.; Matera J.; Miller M. C.; Reuben J. M.; Doyle G. V.; Allard W. J.; Terstappen L. W.; Hayes D. F. Circulating tumor cells, disease progression, and survival in metastatic breast cancer. N. Engl. J. Med. 2004, 351, 781–791. 10.1056/NEJMoa040766. 15317891

[ref2] Danila D. C.; Heller G.; Gignac G. A.; Gonzalez-Espinoza R.; Anand A.; Tanaka E.; Lilja H.; Schwartz L.; Larson S.; Fleisher M.; Scher H. I. Circulating tumor cell number and prognosis in progressive castration-resistant prostate cancer. Clin. Cancer Res. 2007, 13, 7053–7058. 10.1158/1078-0432.CCR-07-1506. 18056182

[ref3] Cohen S. J.; Punt C. J.; Iannotti N.; Saidman B. H.; Sabbath K. D.; Gabrail N. Y.; Picus J.; Morse M.; Mitchell E.; Miller M. C.; Doyle G. V.; Tissing H.; Terstappen L. W.; Meropol N. J. Relationship of circulating tumor cells to tumor response, progression-free survival, and overall survival in patients with metastatic colorectal cancer. J. Clin. Oncol. 2008, 26, 3213–3221. 10.1200/JCO.2007.15.8923. 18591556

[ref4] de Bono J. S.; Scher H. I.; Montgomery R. B.; Parker C.; Miller M. C.; Tissing H.; Doyle G. V.; Terstappen L. W.; Pienta K. J.; Raghavan D. Circulating tumor cells predict survival benefit from treatment in metastatic castration-resistant prostate cancer. Clin. Cancer Res. 2008, 14, 6302–6309. 10.1158/1078-0432.CCR-08-0872. 18829513

[ref5] Scher H. I.; Morris M. J.; Larson S.; Heller G. Validation and clinical utility of prostate cancer biomarkers. Nat. Rev. Clin. Oncol. 2013, 10, 225–234. 10.1038/nrclinonc.2013.30. 23459624 PMC3790270

[ref6] Kelloff G. J.; Sigman C. C. Cancer biomarkers: selecting the right drug for the right patient. Nat. Rev. Drug Discovery 2012, 11, 201–214. 10.1038/nrd3651. 22322254

[ref7] Armstrong A. J.; Luo J.; Nanus D. M.; Giannakakou P.; Szmulewitz R. Z.; Danila D. C.; Healy P.; Anand M.; Berry W. R.; Zhang T.; Harrison M. R.; Lu C.; Chen Y.; Galletti G.; Schonhoft J. D.; Scher H. I.; Wenstrup R.; Tagawa S. T.; Antonarakis E. S.; George D. J.; Halabi S. Prospective Multicenter Study of Circulating Tumor Cell AR-V7 and Taxane Versus Hormonal Treatment Outcomes in Metastatic Castration-Resistant Prostate Cancer. JCO Precis. Oncol. 2020, 1285–1301. 10.1200/PO.20.00200. PMC7608579 33154984

[ref8] van der Toom E. E.; Axelrod H. D.; de la Rosette J. J.; de Reijke T. M.; Pienta K. J.; Valkenburg K. C. Prostate-specific markers to identify rare prostate cancer cells in liquid biopsies.. Nat. Rev. Urol. 2019, 16, 7–22. 10.1038/s41585-018-0119-5. 30479377 PMC6324967

[ref9] Rhim A. D.; Mirek E. T.; Aiello N. M.; Maitra A.; Bailey J. M.; McAllister F.; Reichert M.; Beatty G. L.; Rustgi A. K.; Vonderheide R. H.; Leach S. D.; Stanger B. Z. EMT and dissemination precede pancreatic tumor formation. Cell 2012, 148, 349–361. 10.1016/j.cell.2011.11.025. 22265420 PMC3266542

[ref10] Karabacak N. M.; Spuhler P. S.; Fachin F.; Lim E. J.; Pai V.; Ozkumur E.; Martel J. M.; Kojic N.; Smith K.; Chen P. I.; Yang J.; Hwang H.; Morgan B.; Trautwein J.; Barber T. A.; Stott S. L.; Maheswaran S.; Kapur R.; Haber D. A.; Toner M. Microfluidic, marker-free isolation of circulating tumor cells from blood samples. Nat. Protoc. 2014, 9, 694–710. 10.1038/nprot.2014.044. 24577360 PMC4179254

[ref11] Warkiani M. E.; Khoo B. L.; Wu L.; Tay A. K.; Bhagat A. A.; Han J.; Lim C. T. Ultra-fast, label-free isolation of circulating tumor cells from blood using spiral microfluidics.. Nat. Protoc. 2016, 11, 134–148. 10.1038/nprot.2016.003. 26678083

[ref12] Khoo B. L.; Warkiani M. E.; Tan D. S.; Bhagat A. A.; Irwin D.; Lau D. P.; Lim A. S.; Lim K. H.; Krisna S. S.; Lim W. T.; Yap Y. S.; Lee S. C.; Soo R. A.; Han J.; Lim C. T. Clinical validation of an ultra high-throughput spiral microfluidics for the detection and enrichment of viable circulating tumor cells. PLoS One 2014, 9, e99409 10.1371/journal.pone.0099409. 24999991 PMC4085042

[ref13] Nagrath S.; Sequist L. V.; Maheswaran S.; Bell D. W.; Irimia D.; Ulkus L.; Smith M. R.; Kwak E. L.; Digumarthy S.; Muzikansky A.; Ryan P.; Balis U. J.; Tompkins R. G.; Haber D. A.; Toner M. Isolation of rare circulating tumour cells in cancer patients by microchip technology. Nature 2007, 450, 1235–1239. 10.1038/nature06385. 18097410 PMC3090667

[ref14] Stott S. L.; Hsu C. H.; Tsukrov D. I.; Yu M.; Miyamoto D. T.; Waltman B. A.; Rothenberg S. M.; Shah A. M.; Smas M. E.; Korir G. K.; Floyd F. P. Jr.; Gilman A. J.; Lord J. B.; Winokur D.; Springer S.; Irimia D.; Nagrath S.; Sequist L. V.; Lee R. J.; Isselbacher K. J.; Maheswaran S.; Haber D. A.; Toner M. Isolation of circulating tumor cells using a microvortex-generating herringbone-chip. Proc. Natl. Acad. Sci. U.S.A. 2010, 107, 18392–18397. 10.1073/pnas.1012539107. 20930119 PMC2972993

[ref15] Carter L.; Rothwell D. G.; Mesquita B.; Smowton C.; Leong H. S.; Fernandez-Gutierrez F.; Li Y.; Burt D. J.; Antonello J.; Morrow C. J.; Hodgkinson C. L.; Morris K.; Priest L.; Carter M.; Miller C.; Hughes A.; Blackhall F.; Dive C.; Brady G. Molecular analysis of circulating tumor cells identifies distinct copy-number profiles in patients with chemosensitive and chemorefractory small-cell lung cancer. Nat. Med. 2017, 23, 114–119. 10.1038/nm.4239. 27869802

[ref16] Polzer B.; Medoro G.; Pasch S.; Fontana F.; Zorzino L.; Pestka A.; Andergassen U.; Meier-Stiegen F.; Czyz Z. T.; Alberter B.; Treitschke S.; Schamberger T.; Sergio M.; Bregola G.; Doffini A.; Gianni S.; Calanca A.; Signorini G.; Bolognesi C.; Hartmann A.; Fasching P. A.; Sandri M. T.; Rack B.; Fehm T.; Giorgini G.; Manaresi N.; Klein C. A. Molecular profiling of single circulating tumor cells with diagnostic intention. EMBO Mol. Med. 2014, 6, 1371–1386. 10.15252/emmm.201404033. 25358515 PMC4237466

[ref17] Di Trapani M.; Manaresi N.; Medoro G. DEPArray system: An automatic image-based sorter for isolation of pure circulating tumor cells. Cytometry, Part A 2018, 93, 1260–1266. 10.1002/cyto.a.23687. PMC6590341 30551261

[ref18] Balasubramanian P.; Kinders R. J.; Kummar S.; Gupta V.; Hasegawa D.; Menachery A.; Lawrence S. M.; Wang L.; Ferry-Galow K.; Davis D.; Parchment R. E.; Tomaszewski J. E.; Doroshow J. H. Antibody-independent capture of circulating tumor cells of non-epithelial origin with the ApoStream(R) system. PLoS One 2017, 12, e0175414 10.1371/journal.pone.0175414. 28403214 PMC5389826

[ref19] Le Du F.; Fujii T.; Kida K.; Davis D. W.; Park M.; Liu D. D.; Wu W.; Chavez-MacGregor M.; Barcenas C. H.; Valero V.; Tripathy D.; Reuben J. M.; Ueno N. T. EpCAM-independent isolation of circulating tumor cells with epithelial-to-mesenchymal transition and cancer stem cell phenotypes using ApoStream(R) in patients with breast cancer treated with primary systemic therapy. PLoS One 2020, 15, e0229903 10.1371/journal.pone.0229903. 32214335 PMC7098555

[ref20] Gupta V.; Jafferji I.; Garza M.; Melnikova V. O.; Hasegawa D. K.; Pethig R.; Davis D. W. ApoStream(), a new dielectrophoretic device for antibody independent isolation and recovery of viable cancer cells from blood. Biomicrofluidics 2012, 6, 024133 10.1063/1.4731647. PMC3396706 23805171

[ref21] Luo L.; He Y. Magnetically driven microfluidics for isolation of circulating tumor cells. Cancer Med. 2020, 9, 4207–4231. 10.1002/cam4.3077. 32325536 PMC7300401

[ref22] Kang H.; Kim J.; Cho H.; Han K. H. Evaluation of Positive and Negative Methods for Isolation of Circulating Tumor Cells by Lateral Magnetophoresis. Micromachines 2019, 10, 386 10.3390/mi10060386. PMC6631028 31181790

[ref23] Ozkumur E.; Shah A. M.; Ciciliano J. C.; Emmink B. L.; Miyamoto D. T.; Brachtel E.; Yu M.; Chen P. I.; Morgan B.; Trautwein J.; Kimura A.; Sengupta S.; Stott S. L.; Karabacak N. M.; Barber T. A.; Walsh J. R.; Smith K.; Spuhler P. S.; Sullivan J. P.; Lee R. J.; Ting D. T.; Luo X.; Shaw A. T.; Bardia A.; Sequist L. V.; Louis D. N.; Maheswaran S.; Kapur R.; Haber D. A.; Toner M. Inertial focusing for tumor antigen-dependent and -independent sorting of rare circulating tumor cells. Sci. Transl. Med. 2013, 5, 179ra47 10.1126/scitranslmed.3005616. PMC3760275 23552373

[ref24] Gao D.; Vela I.; Sboner A.; Iaquinta P. J.; Karthaus W. R.; Gopalan A.; Dowling C.; Wanjala J. N.; Undvall E. A.; Arora V. K.; Wongvipat J.; Kossai M.; Ramazanoglu S.; Barboza L. P.; Di W.; Cao Z.; Zhang Q. F.; Sirota I.; Ran L.; MacDonald T. Y.; Beltran H.; Mosquera J. M.; Touijer K. A.; Scardino P. T.; Laudone V. P.; Curtis K. R.; Rathkopf D. E.; Morris M. J.; Danila D. C.; Slovin S. F.; Solomon S. B.; Eastham J. A.; Chi P.; Carver B.; Rubin M. A.; Scher H. I.; Clevers H.; Sawyers C. L.; Chen Y. Organoid cultures derived from patients with advanced prostate cancer. Cell 2014, 159, 176–187. 10.1016/j.cell.2014.08.016. 25201530 PMC4237931

[ref25] Pantel K.; Alix-Panabieres C. Functional Studies on Viable Circulating Tumor Cells. Clin. Chem. 2016, 62, 328–334. 10.1373/clinchem.2015.242537. 26637479

[ref26] Burguillos M. A.; Magnusson C.; Nordin M.; Lenshof A.; Augustsson P.; Hansson M. J.; Elmer E.; Lilja H.; Brundin P.; Laurell T.; Deierborg T. Microchannel acoustophoresis does not impact survival or function of microglia, leukocytes or tumor cells. PLoS One 2013, 8, e64233 10.1371/journal.pone.0064233. 23724038 PMC3664584

[ref27] Magnusson C.; Augustsson P.; Lenshof A.; Ceder Y.; Laurell T.; Lilja H. Clinical-Scale Cell-Surface-Marker Independent Acoustic Microfluidic Enrichment of Tumor Cells from Blood. Anal. Chem. 2017, 89, 11954–11961. 10.1021/acs.analchem.7b01458. 29087172 PMC5698115

[ref28] Cushing K.; Undvall E.; Ceder Y.; Lilja H.; Laurell T. Reducing WBC background in cancer cell separation products by negative acoustic contrast particle immuno-acoustophoresis. Anal. Chim. Acta 2018, 1000, 256–264. 10.1016/j.aca.2017.11.064. 29289318 PMC7437979

[ref29] Augustsson P.; Karlsen J. T.; Su H. W.; Bruus H.; Voldman J. Iso-acoustic focusing of cells for size-insensitive acousto-mechanical phenotyping. Nat. Commun. 2016, 7, 11556 10.1038/ncomms11556. 27180912 PMC4873643

[ref30] Jacob K.; Sollier C.; Jabado N. Circulating tumor cells: detection, molecular profiling and future prospects. Expert Rev. Proteomics 2007, 4, 741–756. 10.1586/14789450.4.6.741. 18067413

[ref31] Gabriel M. T.; Calleja L. R.; Chalopin A.; Ory B.; Heymann D. Circulating Tumor Cells: A Review of Non-EpCAM-Based Approaches for Cell Enrichment and Isolation. Clin. Chem. 2016, 62, 571–581. 10.1373/clinchem.2015.249706. 26896446

[ref32] Pösel C.; Moller K.; Frohlich W.; Schulz I.; Boltze J.; Wagner D.-C. Density gradient centrifugation compromises bone marrow mononuclear cell yield. PLoS One 2012, 7, e50293 10.1371/journal.pone.0050293. 23236366 PMC3516517

[ref33] Johnson L. M.; Gao L.; Shields I. C.; Smith M.; Efimenko K.; Cushing K.; Genzer J.; Lopez G. P. Elastomeric microparticles for acoustic mediated bioseparations. J. Nanobiotechnol. 2013, 11, 22 10.1186/1477-3155-11-22. PMC3706277 23809852

[ref34] Shields C. W. t.; Johnson L. M.; Gao L.; Lopez G. P. Elastomeric negative acoustic contrast particles for capture, acoustophoretic transport, and confinement of cells in microfluidic systems. Langmuir 2014, 30, 3923–3927. 10.1021/la404677w. 24673242

[ref35] Cushing K. W.; Piyasena M. E.; Carroll N. J.; Maestas G. C.; Lopez B. A.; Edwards B. S.; Graves S. W.; Lopez G. P. Elastomeric negative acoustic contrast particles for affinity capture assays.. Anal. Chem. 2013, 85, 2208–2215. 10.1021/ac3029344. 23331264 PMC3621144

[ref36] Grenvall C.; Augustsson P.; Folkenberg J. R.; Laurell T. Harmonic microchip acoustophoresis: a route to online raw milk sample precondition in protein and lipid content quality control. Anal. Chem. 2009, 81, 6195–6200. 10.1021/ac900723q. 19572705

[ref37] Hermanson G. T. From Bioconjugate Techniques, 2nd ed.; Elsevier: Oxford, U.K., 2008; Vol. 204, 205.

[ref38] Augustsson P.; Magnusson C.; Nordin M.; Lilja H.; Laurell T. Microfluidic, label-free enrichment of prostate cancer cells in blood based on acoustophoresis. Anal. Chem. 2012, 84, 7954–7962. 10.1021/ac301723s. 22897670 PMC3445767

[ref39] Nilsson A.; Petersson F.; Jonsson H.; Laurell T. Acoustic control of suspended particles in micro fluidic chips. Lab Chip 2004, 4, 131–135. 10.1039/B313493H. 15052353

[ref40] Gorkov L. P.; Pitaevskii L. P. The Transition of Liquid He-3 into the Superfluid State. Sov. Phys. JETP-USSR 1962, 15, 417–421.

[ref41] Olofsson K.; Hammarstrom B.; Wiklund M. Acoustic separation of living and dead cells using high density medium. Lab Chip 2020, 20, 1981–1990. 10.1039/D0LC00175A. 32356853

[ref42] Yang A. H.; Soh H. T. Acoustophoretic sorting of viable mammalian cells in a microfluidic device. Anal. Chem. 2012, 84, 10756–10762. 10.1021/ac3026674. 23157478 PMC3677785

[ref43] Zalis M. C.; Reyes J. F.; Augustsson P.; Holmqvist S.; Roybon L.; Laurell T.; Deierborg T. Label-free concentration of viable neurons, hESCs and cancer cells by means of acoustophoresis. Integr. Biol. 2016, 8, 332–340. 10.1039/C5IB00288E. 26915333

[ref44] Ley M. W.; Bruus H. Continuum modeling of hydrodynamic particle-particle interactions in microfluidic high-concentration suspensions. Lab Chip 2016, 16, 1178–1188. 10.1039/C6LC00150E. 26948344

[ref45] Urbansky A.; Olm F.; Scheding S.; Laurell T.; Lenshof A. Label-free separation of leukocyte subpopulations using high throughput multiplex acoustophoresis. Lab Chip 2019, 19, 1406–1416. 10.1039/C9LC00181F. 30869100

[ref46] Park S.; Ang R. R.; Duffy S. P.; Bazov J.; Chi K. N.; Black P. C.; Ma H. Morphological differences between circulating tumor cells from prostate cancer patients and cultured prostate cancer cells. PLoS One 2014, 9, e85264 10.1371/journal.pone.0085264. 24416373 PMC3885705

[ref47] Mendelaar P. A. J.; Kraan J.; Van M.; Zeune L. L.; Terstappen L.; Oomen-de Hoop E.; Martens J. W. M.; Sleijfer S. Defining the dimensions of circulating tumor cells in a large series of breast, prostate, colon, and bladder cancer patients. Mol. Oncol. 2021, 15, 116–125. 10.1002/1878-0261.12802. 32949099 PMC7782084

[ref48] Marrinucci D.; Bethel K.; Bruce R. H.; Curry D. N.; Hsieh B.; Humphrey M.; Krivacic R. T.; Kroener J.; Kroener L.; Ladanyi A.; Lazarus N. H.; Nieva J.; Kuhn P. Case study of the morphologic variation of circulating tumor cells. Hum. Pathol. 2007, 38, 514–519. 10.1016/j.humpath.2006.08.027. 17188328

