# Supplementary material for: Two-Step Acoustophoresis Separation of Live Tumor Cells from Whole Blood
Source: Anal Chem. 2021 Dec 16;93(51):17076–85. doi: 10.1021/acs.analchem.1c04050 (PMC8717332; doi:10.1021/acs.analchem.1c04050)
Supplement: Supplementary file 1 — ac1c04050_si_001.pdf [file ac1c04050_si_001.pdf]

Supporting Information

Two-step acoustophoresis separation of live tumor cells from whole blood

Eva Undvall Anand,[a]†
Cecilia Magnusson,[b]†
Andreas Lenshof,[a]
Yvonne Ceder,[c]
Hans Lilja,[b, d,]
Thomas Laurell,[a*]

[a] Department of Biomedical Engineering, Lund University, 221 00 Lund, Sweden.
[b] Department of Translational Medicine, Lund University, 205 02 Malmö, Sweden
[c] Department of Laboratory Medicine, Lund University, 221 00 Lund, Sweden.
[d] Department of Laboratory Medicine, Surgery (Urology), and Medicine (GU Oncology), Memorial Sloan-Kettering Cancer Center, NY 10065, United States.

Correspondance: Thomas Laurell, Box 118, 22100 Lund SWEDEN, thomas.laurell@bme.lth.se, +46462227540

SUPPORTING INFORMATION

1. Supporting Information Experimental Section
2. Supporting Information Figure 1
3. Supporting Information Figure 2

Experimental Section

Synthesis and activation of Biotinylated EPs.

Polydisperse biotinylated elastomeric particles were synthesized as previously described[1]. Briefly, 1 g of a 1:5 mixture of curing agent and Sylgard 184 (Dow Chemical) was emulsified in 10 mL of ultrapure water (Mill-Q, 18MΩ resistivity) containing a biotinylated Pluronic F-108 surfactant. The emulsion was incubated with continuous stirring at 45 ˚C for 90 min and subsequently at room temperature for at least 12 hrs to permit curing. The Pluronic F-108 surfactant was biotinylated following reported protocol[37]. In short, the hydroxyl end groups on Pluronic F108 (Sigma-Aldrich) was modified to disuccinimidyl carbonate with N, N'-disuccinimidyl carbonate (DSC) (Alfa Aesar, Haverhill, MA) and 4-(dimethylamino) pyridine (DMAP) (Merck, Kenilworth, NJ), followed by reaction with biotin hydrazide. Synthesized biotinylated EPs were size fractionated by centrifugation as previously reported with a resulting particle mean diameter of 7.35 μm (SD ±2.86), CV 39.0%[28]. To bind non-fixed WBC, the size fractionated EPs were functionalized with streptavidin conjugated mouse anti-human CD45 monoclonal antibody clone MEM-28 (Sigma-Aldrich) as described[1]. In short, approximately 3 x 10^6 biotinylated EPs were incubated with 1.5 μL streptavidin conjugated anti-CD45 antibody for 1 h at room temperature. The anti-CD45 antibody was previously coupled to streptavidin with a commercially available labeling kit (AbCAM, Cambridge, UK). Following incubation, the particles were washed twice and finally resuspended in FACS buffer (FACS buffer: 1x PBS, 1% FBS, 2 mM EDTA) or FACS buffer containing 0.1% Pluronic F-108 surfactant (FACS buffer P).

Immunostaining, Flow Cytometry enumeration and ImageStream®X analysis.

Cells were incubated with appropriate fluorochrome-conjugated antibody according to manufacturer recommendation or with isotype control IgG_1-antibodies for 25 min at room temperature and subsequently washed twice with Dulbecco's Phosphate Buffered Saline (DPBS; Sigma-Aldrich) and re-suspended in FACS buffer. Samples of 0.5 – 1.0 mL RBC lysed blood spiked with 1,000-10,000 DU145 cells were stained with epithelial cell marker anti-EpCAM-APC or anti-EpCAM-PE clone EBA-1 (BD Biosciences). WBCs were labeled with anti-CD45-APC clone HI30 (BD Biosciences) for 25 min at room temperature in enumeration experiments when EPs were not present. Cells were run on FACS Canto II (BD Biosciences) with the BD FACSDiva™ software for cell analysis and enumeration. Cancer cells (DU145) were gated as $EpCAM^+$ and $CD45^-$. Amnis ImageStream®X Mk II (Millipore, Burlington, MA) was used to obtain images of elastomeric particle complexes with captured white blood cells stained with DAPI (Sigma-Aldrich) for 5 min (on ice) and of isolated DU145 cells stained with anti-EpCAM-APC, and the IDEA® analysis software was used for image analysis. DAPI was used to stain WBCs when EPs were present, as the CD45 surface marker normally used for WBC staining was also used by the EPs.

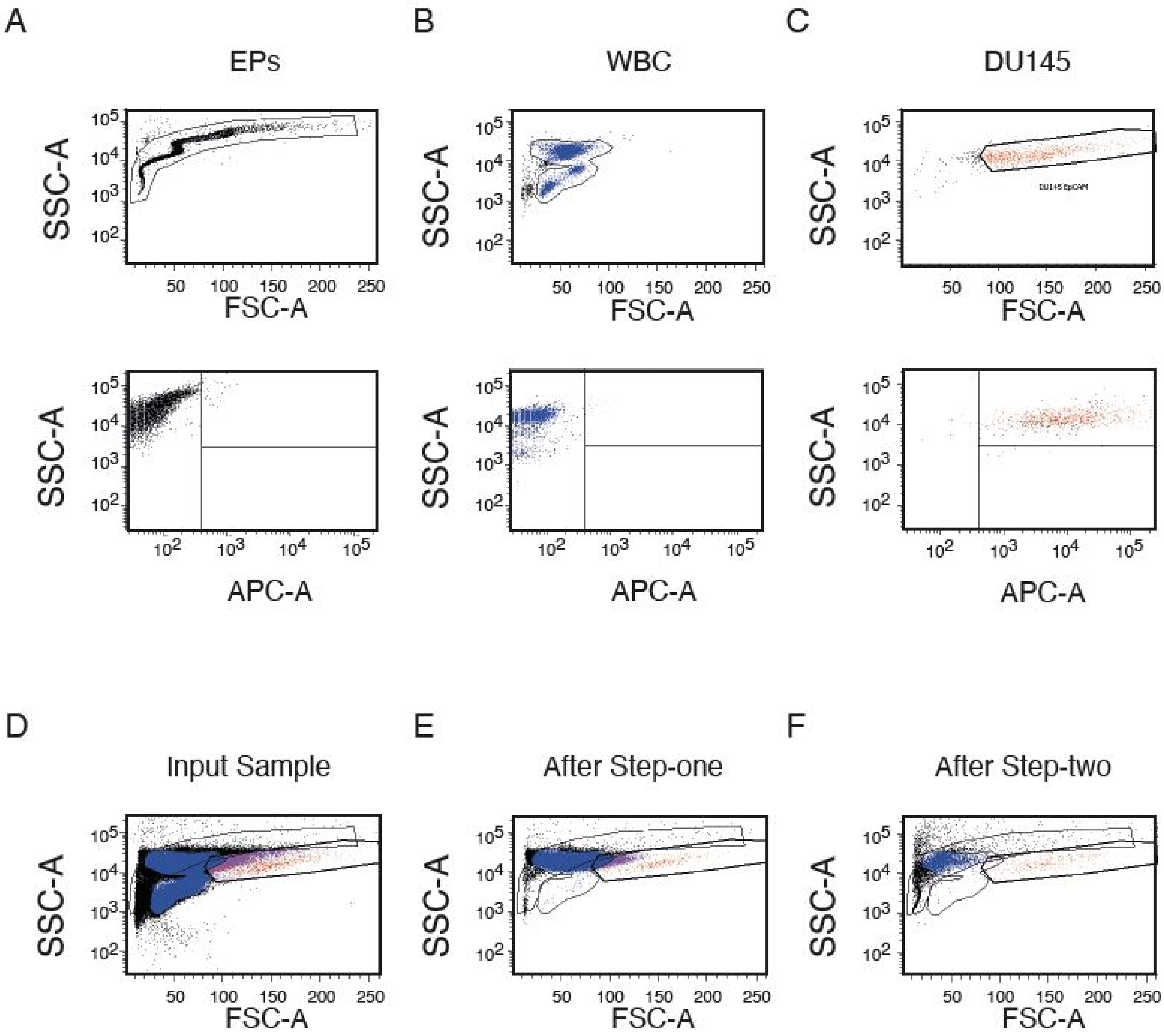

Figure S1. Flow cytometry gating strategy for concentration measurement of EPs, WBC and DU145 cells, and for output sample measurements. A) The characteristic spiral-like population of EPs (black dots) in FSC-A *vs*. SSC-A (upper panel), with absence of APC-A signal (lower panel). B) WBC (blue dots) gating in FSC-A *vs*. SSC-A (upper), with absence of APC-A signal (lower). C) DU145 (red dots) cell gating in FSC-A *vs*. SSC-A (upper), with a positive APC-A signal (lower) from the bound EpCAM-APC complex. D) Representative FSC-A *vs*. SSC-A plot for a 1 mL input sample with 1,000 DU145 cells spiked into RBC lysed whole blood mixed 1:1 with FACS buffer. To obtain reference values for the input sample, the entire 1 mL sample was analyzed and not used for further A^2 enrichment. E) Representative FSC-A *vs*. SSC-A plot of a 1 mL enriched output sample after the primary separation step, where the lymphocytes and monocytes and most of the granulocytes were removed (5-10% remaining). Again, the entire 1 mL sample was analyzed. F) Representative FSC-A *vs*. SSC-A plot over a 1 mL output sample after the entire A^2 separation. For all flow cytometry analyses BD FACS CANTOII and FACS Diva software from BD Biosciences was used. The defined cell gating strategies were applied for analysis.

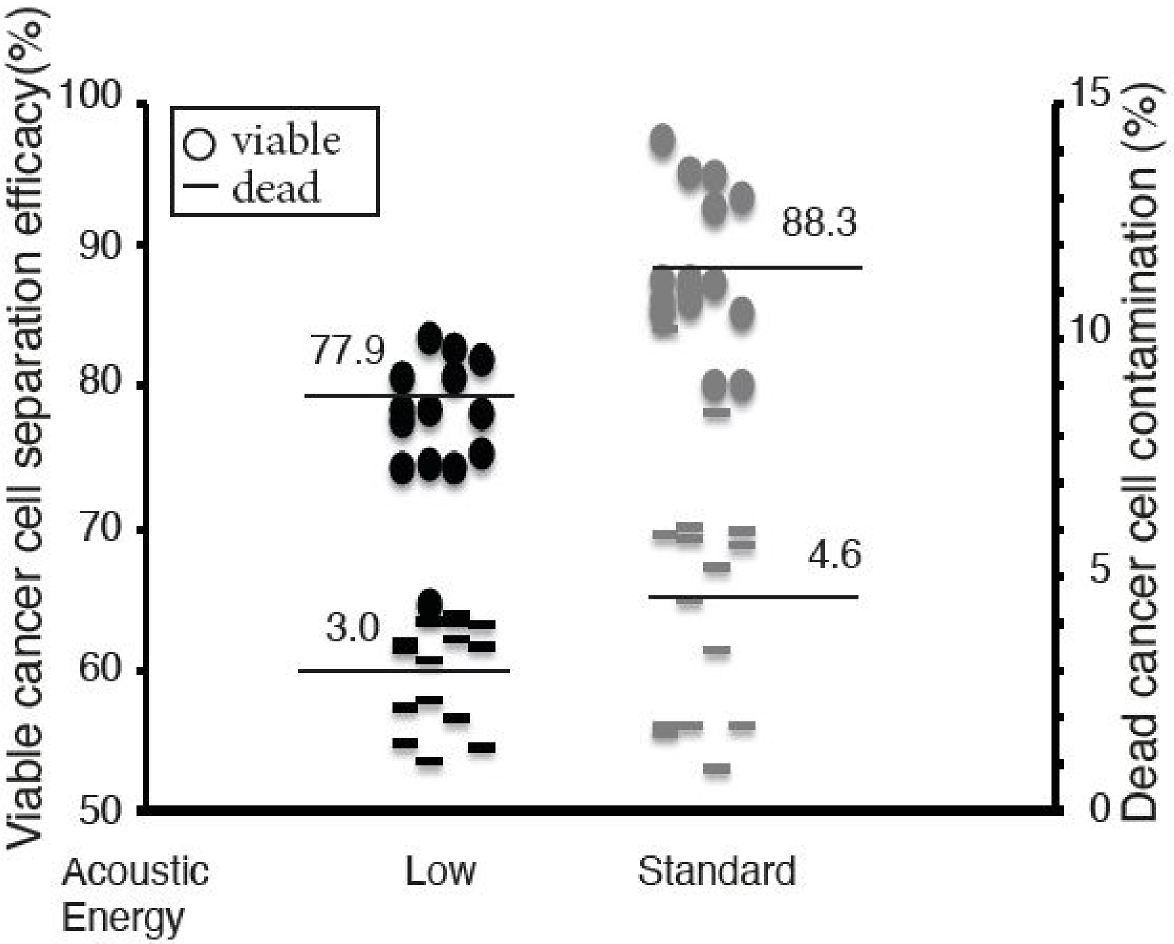

Figure S2. Separation of viable and dead cells by acoustophoresis. The graph shows percentage viable (circle) DU145 cell recovery and dead (dash) cells contamination at the central outlet compared to the side outlet, after acoustic cell separation at two different acoustic energy levels.

1. Cushing, K.; Undvall, E.; Ceder, Y.; Lilja, H.; Laurell, T., Reducing WBC background in cancer cell separation products by negative acoustic contrast particle immuno-acoustophoresis. *Anal Chim Acta* **2018,** *1000*, 256-264.

2. Hermanson, G. T., *From Bioconjugate Techniques*. 2nd ed.; Elsevier: Oxford, UK, **2008**; Vol. 204, 205.
